# Aerobic vaginitis in the third trimester and its impact on pregnancy outcomes

**DOI:** 10.1186/s12884-022-04761-5

**Published:** 2022-05-24

**Authors:** Anh Thi Chau Nguyen, Na Thi Le Nguyen, Thu Thi Anh Hoang, Tuyen Thi Nguyen, Trang Thi Quynh Tran, Dan Nu Tam Tran, Anh Thi Kim Nguyen, Linh Manh Tran, Duc Huu Chau Nguyen, Tam Minh Le, Binh Duy Ho, Tiiu Rööp, Siiri Kõljalg, Jelena Štšepetova, An Van Le, Andres Salumets, Reet Mändar

**Affiliations:** 1 Department of Microbiology, Hue University of Medicine and Pharmacy, Hue University, 06 Ngo Quyen Street, Hue, Vietnam; 2 Becamex International Hospital, Thuan An City, Binh Duong, Vietnam; 3 Hue University of Medicine and Pharmacy, Hue University, 06 Ngo Quyen Street, Hue, Vietnam; 4 Department of OBGYN, Hue University of Medicine and Pharmacy, Hue University, 06 Ngo Quyen Street, Hue, Vietnam; 5 Center for Reproductive Endocrinology and Infertility, Hue University of Medicine and Pharmacy, Hue University, 06 Ngo Quyen Street, Hue, Vietnam; 6 Department of Microbiology, Institute of Biomedicine and Translational Medicine, University of Tartu, 50411 Tartu, EE Estonia; 7 Institute of Clinical Medicine, Department of Obstetrics and Gynecology, University of Tartu, 51014 Tartu, Estonia; 8 Division of Obstetrics and Gynecology, Department of Clinical Science, Intervention and Technology (CLINTEC), Karolinska Institutet, Stockholm, Sweden; 9 Competence Centre On Health Technologies AS, 50410 Tartu, Estonia

**Keywords:** Aerobic vaginitis, Pregnancy, Puerperal sepsis, Neonatal infections

## Abstract

**Background:**

Aerobic vaginitis (AV) is a vaginal inflammation characterized by disruption of the lactobacillus microbiota and increased counts of different aerobic bacteria. AV may result in severe complications, especially during pregnancy, including preterm delivery, neonatal and maternal infections. This study aimed to determine the prevalence of AV in the third trimester of pregnancy, and the relationship between AV and pregnancy outcomes.

**Methods:**

A cross-sectional descriptive study included 323 pregnant women attending for routine antenatal care in the Hue University Hospital. Vaginal samples collected at the third trimester of pregnancy were evaluated for AV according to the scoring system of Donders and cultured for identification of predominant bacteria. Pregnancy was followed to its end, and pregnancy outcomes were recorded for both mothers and infants.

**Results:**

The proportion of pregnant women diagnosed with AV in the third trimester was found to be 15.5%, with the vast majority of the cases (84%) displaying the light AV and 16% the moderate AV. The vaginal cultures in the women with AV revealed most frequently *Streptococcus agalactiae* (6%), followed by *Enterococcus* spp (4%), *Staphylococcus aureus* (4%), and *Acinetobacter baumannii* (2%). In addition, AV during the last trimester of pregnancy was associated with an increased risk of puerperal sepsis (OR 8.65, 95% CI: 1.41—53.16, *p* = 0.020) and there was a slightly increased risk for neonatal infections, which was statistically insignificant.

**Conclusions:**

The proportion of AV is relatively high in Vietnamese pregnant women. Since it is associated with an increased risk of puerperal sepsis, it needs to be diagnosed and treated before delivery.

## Background

In 2002, the term “aerobic vaginitis” (AV) was first identified by Donders et al. [1]. AV is described as a depletion of the *Lactobacillus* microbiota and an increase in aerobic bacteria derived mainly from the gastrointestinal tract, accompanied by inflammatory markers and deficient epithelial maturation (Table 1). AV is characterized by the presence of several aerobic and facultative bacteria, such as *Streptococcus agalactiae*, *Enterococcus faecalis*, *Escherichia coli*, coagulase negative staphylococci (CoNS) and *Staphylococcus aureus* [2]. Clinical presentation includes purulent homogenous discharge (yellow or yellow-green), foul-smelling rotten odor, introital and vaginal redness (in case of severe AV, also ecchymotic bleeding points and erosions), stinging and burning sensations, and dyspareunia. Symptoms can be present for a long period of time and fluctuate in terms of severity [3].

**Table 1:** Criteria for the microscopic diagnosis of aerobic vaginitis (AV) by Donders et al. [1]

**AV score**	**Lactobacillary grades (LBG)**	**Number of leukocytes**	**Proportion of toxic leukocytes**^b^	**Background flora**	**Proportion of parabasal epitheliocytes**^a^
0	I and IIa LBG I – numerous pleomorphic LB, no other bacteria LBG IIa – mixed flora, but predominantly LB	≤ 10/hpf	None or sporadic	Unremarkable or cytolysis	< 1%
1	IIb Mixed flora, but proportion of LB severely decreased due to increased number of other bacteria	> 10/hpf and ≤ 10/epithelial cell	≤ 50% of leukocytes	Small coliform bacilli	< 10%
2	III LB severely depressed or absent because of overgrowth of other bacteria	> 10/ epithelial cell	> 50% of leukocytes	Cocci or chains	≥ 10%

Total score of < 3, no signs of AV; score 3–4, light AV; score 5–6, moderate AV; score 7–10, severe AV (the latter group resembles ‘desquamative atrophic vaginitis’)

*Hpf* High-power field (400 × magnification), *LB* Lactobacilli, *LBG* Lactobacillary grade

^a^Parabasal epitheliocytes – small round immature epithelial cells with a high nuclear to cytoplasmic ratio

^b^Toxic leucocytes – swollen leucocytes containing granules showing lysosomal activity

AV has been observed in approximately 4–8% of pregnant women [3] and in 5–24% of women complaining of abnormal vaginal discharge [2]. To date, AV has not yet been considered a specific concern for pregnancy. It is often mistakenly diagnosed with bacterial vaginosis (BV), an anaerobic imbalance of vaginal microbiota. Meanwhile, between AV and BV, there are numerous distinctive characteristics of clinical symptoms like type of vaginal discharge, Whiff test results, presence of inflammation, level of immune reaction and different cellular pattern. Diagnostic confusion can lead to treatment disappointment and has been related to serious pregnancy complications such as preterm birth, premature rupture of membranes (PROM), miscarriage, chorioamnionitis and neonatal infections [3–6]. At the same time, preterm birth and neonatal infection are two of the top three causes of neonatal mortality (along with neonatal asphyxia). Less is known about the association of AV with puerperal sepsis, though this is one of the top five causes of maternal deaths worldwide.

Screening for vaginitis during pregnancy is prescribed routinely at the time of 12–16 weeks of pregnancy in most countries. However, in Vietnam, there is no scheduled testing for pregnant women, especially those who do not have symptoms that indicate infection. This may lead to the above-mentioned obstetric risks and increase the rate of admission to the neonatal intensive care unit (NICU). For the above reasons, we carried out the study in order to reveal the proportion of women having AV during the last trimester of pregnancy. In addition, we also aimed to look for the relationship between AV and pregnancy outcomes.

## Methods

### Study subjects

Participants for this cross-sectional descriptive study were recruited among pregnant women who attended routine antenatal care during the last trimester of pregnancy in the Hue University Hospital, Vietnam, from July 2018 until January 2019. All the women had live fetuses without fetal anomalies. The exclusion criteria were previously diagnosed rupture of membranes, antepartum hemorrhage, vaginal douching before or during vaginal specimen collection; those who had been treated for reproductive tract infections or who had used antibiotics for some other reasons within the last week.

The sample size was calculated according to the prevalence of pregnant women in the last trimester carrying microorganisms that cause lower genital tract diseases, according to the previous research from the Hue University Hospital (42.9%) [7]. With 95% CI and 0.16 of relative deviation, our study needed the minimum sample size of 200 cases.

This study was approved by the Ethics Committee of Hue University of Medicine and Pharmacy, Vietnam (No. H2018/162 dated May 24th, 2018). All study participants accepted and signed the consent form. Participation in the study was voluntary. Written informed consent was obtained from all study subject. All methods were performed in accordance with the relevant guidelines and regulations, including Declaration of Helsinki.

### Clinical investigations and sample collection

Pregnant women in the last trimester of pregnancy (≥ 28 weeks) eligible for treatment and/or labor at the Hue University Hospital were asked for general information, clinical examination and sampling. Background data included age, education, profession, marital status, and history of reproductive health (genital tract infections, abortions, and miscarriages).

The women were monitored until delivery, and both maternal and neonatal outcomes were recorded. Clinical data of the pregnant women included PROM, preterm premature rupture of membranes (pPROM), ﻿chorioamnionitis (an acute inflammation of the membranes and chorion of the placenta), ﻿delivery mode, and puerperal health. Puerperal sepsis was defined as the infection of the genital tract occurring at labour or within 42 days of the postpartum period, in which two or more of the following are present: pelvic pain, fever, abnormal foul-smelling vaginal discharge, delay in the reduction of the uterine size. Clinical data of the newborns included birth weight, Apgar scores (comprising five components: color, heart rate, reflexes, muscle tone, respiration), and neonatal infections.

Vaginal samples were collected mostly (91%) after 37 weeks of pregnancy. Vaginal fluid was taken from the vagina, at the site of suspected infection or at the posterior vaginal fornix. Two sterile cotton swabs were used for sampling. The swabs were transported to the Department of Microbiology in the Hue University Hospital of Medicine and Pharmacy within 2 h or stored at 4 °C within 12 h before sending to the lab. At the lab, one swab was used for making Gram stained slide smear, the second swab was frozen in cryotube (with BHI broth and 20% glycerol) for the subsequent cultures in University of Tartu, Estonia.

### Laboratory methods

The microscopic AV diagnostic criteria proposed by Donders et al. were applied under microscope applying both 400 × and 1000 × magnification [3, 8, 9]. Lactobacillary grades, other bacteria, leukocytes and parabasal epithelial cells were recorded. AV was diagnosed as indicated in Table 1. AV was classified as light AV (score 3 or 4), moderate AV (score 5 or 6) and severe AV (score ≥ 7).

In addition, vaginal fluid was cultured onto blood agar media, the cultures were incubated for 18–24 h in aerobic conditions. The predominant colonies were counted and identified by MALDI-TOF MS (Bruker Daltonics) at the Department of Microbiology in University of Tartu, Estonia.

### Statistical methods

The collected data were stored in MS Excel 2016. SPSS 20.0 software was used for analysis and processing the data. Factors related to AV were determined to have statistically significant differences if *p* < 0.05.

## Results

### Clinical characteristics of the study subjects

In total 323 cases were eligible to be enrolled in the study (Table 2). Their mean age was 28.3 ± 5.1 years, 64.1% of them belonged to age group 25–34 years. Most of the women had education at the secondary school level and above. In term of career, public servants and housewives accounted for the majority. Vast majority of women were married, while only 0.6% of respondents were single. The proportion of pregnant women with a history of lower genital tract infections accounted for 18.9%, and that of with previous abortion or miscarriage 20.7%.

**Table 2:** Background characteristics of the study group (*n* = 323)

Variable	N	Rate (%)
**Age (years)**		
< 18	3	0.9
18—24	75	23.2
25—34	207	64.1
≥ 35	38	11.8
Average age (years)	28.3 ± 5.1	
**Profession**		
Public servants	133	41.2
Agriculture-forestry-fishery	13	4.0
Housewife	92	28.5
Business	50	15.5
Other	35	10.8
**Education level**		
Illiteracy	0	0.0
Primary school	9	2.8
Secondary or high school	222	68.7
College or above	92	28.5
**Marital status**		
Married	321	99.4
Unmarried	2	0.6
**Sampling time (week of pregnancy)**		
< 37	30	9.3
≥ 37	293	90.7
**History of lower genital tract infections**	61	18.9
**History of abortion or miscarriage**	67	20.7

### Prevalence and impact of aerobic vaginitis

The prevalence of AV in Vietnamese pregnant women made up 15.5%, most of these women (42 out of 50) had light AV and 8 women had moderate AV (Table 3). The cultures revealed different aerobic and facultative bacteria in these women, most frequently *Streptococcus agalactiae* (6%), *Enterococcus* spp (4%) and *Staphylococcus aureus* (4%), followed by *Acinetobacter baumannii* (2%) (Table 4). The cultures in the women without AV revealed most frequently enterococci (6.7%).

**Table 3:** AV status in the investigated pregnant women (*n* = 323)

AV status	N	Rate (%)
No signs of AV	273	84.5
AV		
Light (score 3–4)	42	13.0
Moderate (score 5–6)	8	2.5
Severe (score 7–10)	0	0
**Total**	**323**	**100**

**Table 4:** Results of the vaginal cultures in the pregnant women having AV (*n* = 50)

Culture result of vaginal fluid	N	Rate (%)
Negative	42	84.0
Positive		
* Acinetobacter baumannii*	1	2.0
* Enterococcus* spp	2	4.0
* Staphylococcus aureus*	2	4.0
* Streptococcus agalactiae*	3	6.0
**Total**	**50**	**100**

AV was associated with an increased risk of postpartum infection, the puerperal sepsis (OR 8.65, 96% CI: 1.4–53.2, *p* = 0.020) (Table 5). Slightly increased risk for neonatal infections was also seen, but this difference (16.0% for AV vs. 10.3% for non-AV pregnancies) was not statistically significant.

**Table 5:** Association of pregnancy outcome with AV (*n* = 323)

Variable	AV present (*n* = 50)	No AV (*n* = 273)	OR (95% CI)	p
	N (%)	N (%)		
**Preterm labor**	1 (2.0)	9 (3.3)	0.60 (0.07–4.83)	0.631
**Rupture of membranes:**				
** PROM**^**a**^	15 (30.0)	91 (33.3)	0.86 (0.45–1.65)	0.645
** pPROM**^**a**^	0 (0.0)	2 (0.7)	0.68 (0.03–14.46)	0.807
**Chorioamnionitis**	0 (0.0)	0 (0.0)	-	-
**Childbirth:**				
** Caesarean section**	26 (52.0)	152 (55.7)	0.86 (0.47–1.58)	0.631
** Vaginal labor**	24 (48.0)	121 (44.3)	-	-
**Puerperal sepsis**	3 (6.0)	2 (0.7)	8.65 (1.41–53.16)	**0.020**
**Low birth weight (< 2500 g)**	**1 (2.0)**	**11 (4.0)**	**0.49 (0.06–3.85)**	**0.495**
**Apgar score:**				
** Apgar score 1 min < 8**	0 (0.0)	2 (0.7)	1.08 (0.05–22.73)	0.963
** Apgar score 5 min < 8**	0 (0.0)	1 (0.4)	1.80 (0.07–44.78)	0.720
**Neonatal infections**	8 (16.0)	28 (10.3)	1.71 (0.73–4.00)	0.219

*PROM* Premature rupture of membranes, *pPROM* preterm premature rupture of membranes

^a^Revealed during sample collection

## Discussion

Our data showed that 15.5% of Vietnamese pregnant women had AV in the third trimester of pregnancy, and majority of these cases represented AV light form (13%), while the moderate AV was diagnosed only in 2.5% of pregnant women. However, despite of the light/moderate form, the AV was significantly associated with an increased risk of postpartum infections that is considered to be one of the leading causes of maternal death worldwide.

### Etiopathogenesis, diagnosis and treatment of AV

Healthy vaginal microbiota is dominated by lactobacilli while imbalance of this community may result in AV, BV or candidiasis. In case of AV, lactobacilli counts are decreased while that of several enteric bacteria are increased, including *S. agalactiae*, *E. faecalis*, *E. coli*, CoNS and *S. aureus* [2]*.* During last years, some studies have applied next-generation sequencing to AV patients. These studies have indicated increased number of species [10], elevated proportions of *Actinobacteria* and *Bacteroidetes* [11], and surprisingly also high abundance of *Gardnerella vaginalis* and *Prevotella bivia,* in addition to that of *S. agalactiae* [8]. Not only the species composition, but also their microbial concentration may be of importance. Moreover, it is not finally clear whether this imbalanced community is of the causative factor of the AV or the associated feature present in AV. It has also been proposed that AV may be an immunological disorder with an influence on the vaginal microbiota [12]. This idea is supported by variable degrees of inflammation, not only measured by the number of leucocytes, but also by the proportions of so-called ‘toxic leucocytes’ (swollen leucocytes containing granules showing lysosomal activity), imbalance of other immune markers and a variable presence of parabasal or immature epithelial cells. Other possible pathogenetic factors include low local estrogen level, presence of sialidase-producing bacteria and deficiency of vitamin D [3], while also presence of some sexually transmitted infections [4] has also been linked to AV. Intrauterine devices, being unmarried, long term use of antibiotics and frequent vaginal douching have been considered to contribute to the AV etiology [13].

Diagnosis of AV can be made using wet mount phase contrast microscopy. An AV score is calculated according to lactobacilli grade (LBG), presence of inflammation, proportion of toxic leukocytes, microbiota characteristics, and parabasal epithelial cells. A score ranging from 0 to 2 is assigned to each of the above-mentioned characteristics, and AV is diagnosed according to the composite score: 3–4 indicates light AV, 5–6 represents moderate AV and 7–10 represents severe AV (Table 1). Cultures can give additional information about the microbiota composition but the diagnosis cannot rely on the cultures only. To circumvent the hurdle of microscopic investigation, some attempts have been made to develop nucleic-acid-based and enzymatic diagnostic tests, but the detailed information obtained with microscopy, especially cellular pattern, has remained irreplaceable [3, 14]. The AV diagnostic criteria among previous studies have not been unanimous. Choice of diagnostic approach depends on the suspected diagnosis and the readiness of the health care system to recognize, diagnose and treat AV. Clinical picture is often nonspecific, affecting the initial diagnostic approach as well as prescribing inaccurate medication. Most clinicians deem that the expected AV rate may be higher and that the majority of AV cases remain undiagnosed. Though, many researchers and clinicians increasingly focus on AV in case of vaginitis symptoms. Majority of studies have used Gram stain microscopy to reveal lactobacilli and other bacteria, rate of leukocytes and presence of parabasal epitheliocytes [15, 16]. We have applied similar diagnostic method on the group of Vietnamese pregnant women. In addition to microscopy, we performed also cultures that revealed common list of bacteria that is characteristic to AV. *S. agalactiae,* enterococci and *S. aureus* were the three predominant bacteria in our AV patients being somewhat different from the previous studies [6, 9], where *E. coli* made up 80—90% of AV cases in pregnant women. To date, there is not much information on the consistency between AV diagnostic criteria by microscopy and culture, thus explaining the discrepancies between the results of the current and previous studies.

One component of the AV-complex, the group B streptococci (GBS) or *S. agalactiae* is a common causative agent of neonatal infections, therefore routine screening of this species at the end of pregnancy has been recommended [17–19]. Its prevalence is about 10–30% being fairly higher than the reported AV during pregnancy in the present study. It must be pointed that the presence of this species does not mean AV diagnosis. Our previous study revealed 6% and 9.5% when a culture or SYBR Green real-time PCR technique was applied to 116 pregnant women to detect GBS, respectively [20]. In the present study, culture revealed this species from 10 women (3%) from the 323 cohort of pregnant women.

The best treatment for AV patients is not yet fully determined, but it must be tailored according to the microscopic findings and the patient's needs. There is a role for estrogen therapy, corticosteroids, local antiseptics, antibiotics (clindamycin, moxifloxacin, carbapenems, combinations of beta-lactam with beta-lactamase inhibitor and others) and probiotics, depending on the leading symptoms and comorbidity. Metronidazole that is commonly used to treat BV is not effective in case of AV [3]. It cannot be forgotten that AV can co-occur with other genital tract infections, such as BV, candidiasis and sexually transmitted infections, therefore these diseases need to be ruled out in AV patients.

### Prevalence of AV in pregnancy

The prevalence of AV is largely unknown, both in pregnant and unpregnant women, since routine diagnostics in case of vaginitis includes detection of BV, candidiasis and trichomoniasis but not AV. Since reliable tests are not commercially available and wet mounts are not routinely performed in many settings, the AV is likely underreported. Therefore, it has been recommended to include AV in the diagnostic workup for patients seeking medical care for vaginitis symptoms [10].

In our study, the prevalence of AV in late pregnancy was 15.5%, mainly representing the light form of the disease (13.0%) and lesser extent (2.5%) the moderate form of AV. No severe cases were found in our study. These numbers are generally comparable to the previous reports where the prevalence of AV has been reported to be 4–8% in pregnant women and 7–13% in non-pregnant women [3]. Hence, the AV is less common than BV, and it tends to be slightly less represented among pregnant than non-pregnant women, being probably associated with physiological changes in the vaginal environment. However, according to some studies the AV in pregnant women may be even higher in prevalence than in non-pregnant women [21]. Still, the available data is not yet sufficient to draw the final conclusions, and moreover, the used methods have varied between the studies.

### Impact of AV on pregnancy outcome

Previous studies have revealed that AV can be related to different pregnancy complications like chorioamnionitis, fetal infection, premature birth, PROM and pPROM as well as neonatal infections [3, 4], less data is available about postpartum infections [4]. Thus, the profile of complications tends to be quite similar to that of BV.

In our study, 6% of pregnant women diagnosed with AV had a puerperal sepsis, whereas in the group without AV, the rate was only 0.7%. Thus, the AV patients had almost nine-fold increased risk (OR = 8.65) of postpartum infection compared to the controls. Postpartum infections account for a significant, and often preventable, portion of the global healthcare burden. They are one of the top five causes of maternal deaths worldwide and account for 10–15% of deaths in the postpartum period. Puerperal sepsis causes more than 75,000 maternal deaths annually [22]. Abnormal vaginal microbiota forms an important reservoir for causative agents of these infections. This is a strong indication that AV needs to be diagnosed and treated in pregnant women. The reasons why AV can be associated with different neonatal and maternal adverse pregnancy outcomes are not finally known but the bacterial pattern and load may likely have its impact.

The most prevalent bacteria in our AV patients were *S. agalactia*e, enterococci and *S. aureus*, which may cause puerperal and neonatal infections. The frequency of other pregnancy complications and caesarean section was not different in women with and without AV in our study, and the preterm birth rate was low both groups (2% vs 3.3%). Yet, to the contrary of the current study, some previous studies have revealed association of AV with preterm labor and PROM [3, 4]. In our research, we analyzed the rate of amniotic fluid rupture at all the gestational ages, without seeing the effect of AV on either PROM or pPROM.

Even though screening and prevention policies for *S. agalactiae* have been systematically introduced, early neonatal infection caused by this species continues to be a problem. However, since the turn of the millennium, the neonatal infections have remained on the plateau [19]. The screening and prevention of neonatal Group B streptococcus infections have currently been primarily successfully applied in term infants, while the current pattern of neonatal infection and mortality caused by *S. agalactiae* has still remained the serious risk factor for preterm neonates [17]. In our study, both groups had similar preterm birth rates, but prevalence of *S. agalactiae* was only 3% in the full cohort, being lower than in the general reports. In the current study, the neonatal sepsis was found in 36 neonates (11.1% out of all 323 neonates), while the neonatal sepsis occurred slightly, but insignificantly more frequently in AV (16%) than in non-AV (10.3%) groups of pregnant women. Still, we have no data and solid clue about the etiology of the neonatal infections in our study groups.

Our study has several limitations. Majority of pregnant women were admitted to our hospital during the final weeks before delivery, therefore we have no information about the previous weeks. The women did not get treatment for AV, hence we have no data about the possible effect of treatment. Since the puerperal sepsis was recorded according to clinical criteria and by using stain method, we have no information about the causative agents. In addition, the study was not adequately powered to find outcome differences such as puerperal or neonatal sepsis, instead, the sample size was calculated for detecting the prevalence of AV in pregnant women.

## Conclusions

The proportion of Vietnamese pregnant women diagnosed with AV in the third trimester accounts for 15.5% of all pregnant women, with the vast majority of cases representing the light disease form. Pattern of cultured bacteria includes common intestinal aerobes with *S. agalactiae, Enterococcus* sp and *S. aureus* being the most prevalent. AV in pregnant women is significantly associated with an increased risk of postpartum infections and therefore needs to be diagnosed and treated before delivery.

## Data Availability

The datasets used and analysed during the current study are available from the corresponding author on reasonable request.

## Abbreviations

AV: Aerobic vaginitis
BV: Bacterial vaginosis
CoNS: Coagulase negative staphylococci
GBS: Group B streptococci
LBG: Lactobacilli grade
NICU: Neonatal intensive care unit
PROM: Premature rupture of membranes
pPROM: Preterm premature rupture of membranes
